# Role of MAPKs in HSP70's Protection against Heat Stress-Induced Injury in Rat Small Intestine

**DOI:** 10.1155/2018/1571406

**Published:** 2018-07-12

**Authors:** Yue Hao, Yuejin Feng, Jielei Li, Xianhong Gu

**Affiliations:** State Key Laboratory of Animal Nutrition, Institute of Animal Sciences, Chinese Academy of Agricultural Sciences, Beijing 100193, China

## Abstract

**Aim:**

To evaluate the role of heat shock protein 70 (HSP70) on the MAPK pathway activation with quercetin treatment and its protection against small intestine impairments of heat stressed rats.

**Methods:**

Forty-eight male Sprague-Dawley rats aged 6 weeks were randomized to three groups (n=16/group), namely, control (CON), heat stress (HS), and heat stress + quercetin (HQ). The experiment lasted for 14 days with daily 50 min of heat stress treatment (43°C) for the HS and HQ groups. Rats of HQ group were intragastrically given 0.5 ml quercetin solution (50 mg/kg body weight) before the heat stress treatment. Half of the animals were sacrificed on day 7 and the rest on day 14 for tissue sampling. Intestinal morphology, small intestine morphology and permeability, protein expression of HSP70, phosphorylation of extracellular signal-regulated kinase (ERK), c-Jun N-terminal kinase (JNK), p38 mitogen-activated protein kinase (MAPK), and caspase-3 activity were examined.

**Results:**

Heat stress caused morphological damage to the small intestine and increased intestinal permeability. HSP70 expression and MAPK activity in the small intestine were increased by heat stress. Inhibition of HSP70 by quercetin did not change intestinal permeability compared with the HS group but aggravated intestinal injury and affected the activation of MAPKs and caspase-3.

**Conclusions:**

HSP70 may modulate stress-activated signaling and acts in a protective manner via MAPK signaling. Affecting HSP70 protective mechanisms could be useful for protection against heat stress-induced injury in rat small intestine.

## 1. Introduction

As a result of global warming, heat stress has become one of the main environmental stressors that negatively affects animal performance, health, and economy [1, 2]. Intestine plays important roles in terms of barrier, digestion and absorption of nutrients, and immunity [3–6]. The gastrointestinal tract is particularly responsive to stressors, especially hyperthermia, which can negatively affect the normal, protective microbiota [7] and the integrity of intestinal epithelium [2, 8]. It has been evidenced that heat stress induces significant intestinal damage [3, 6].

When living organisms are exposed to thermal stress, the synthesis of most proteins is delayed, but a group of highly conserved proteins known as heat shock proteins or heat stress proteins (HSPs) are rapidly synthesized [9]. HSPs play an important role in the survival of stressed cells and the stabilization of the internal environment [10]. HSPs, in general, are involved in normal protein folding, correction of misfolded proteins, and degradation of damaged proteins [11]. The family of HSP70 proteins is one of the most conserved and important members among HSPs [12, 13]. HSP70 is involved in modulating a variety of physiological processes, including stress responses, proliferation, and apoptosis. In addition, HSP70 regulates apoptotic signaling pathways in different manners, such as promoting or suppressing apoptosis [11]. In terms of apoptosis, there are two key players, the transcription factors Bcl-2 and p53, whose expression can be affected by HSP70 [14]. HSP70 also participates in the regulation of myocardial apoptosis after ischemia-reperfusion injury, neurodegeneration, and chronic diseases (e.g., diabetes and neurological disorders) [15]. The early steps of organ regeneration were reported to be primarily regulated by HSP70 [16].

It has been shown that cells respond to elevated temperature exposure by activation of p38 MAPK, ERK1/2, and JNK, as well as by increasing the production of HSPs such as HSP27, HSP40, and HSP70 [17–19]. Furthermore, recent studies reported that there was a correlation between protective HSP70 expression and activation of the MAPK pathway [20, 21]. Previous results indicate that the ERK- or p38 MAPK-mediated induction of HSP70 might play a crucial role in inhibiting caspase-3 activation [20, 22, 23]. However, to the best of our knowledge, the exact role of HSP70 in MAPK signaling pathways under heat stress remains unknown, especially in animal models.

Quercetin (3,3′,4′,5,7-pentahydroxyflavone) is a naturally occurring flavonoid and is known to inhibit HSP70 induction [24]. Quercetin induces apoptosis in* X. laevis* A6 cells through the downregulation of HSPs [25]. In cancer cells, quercetin enhances the cytotoxic effect of paclitaxel through the regulation of HSP70, but the mechanism remains unknown [26]. A study showed that quercetin improved the efficacy of radiofrequency ablation by inhibiting HSP70 [27]. Quercetin inhibits the HSP70-mediated protective effects against endoplasmic reticulum stress-induced apoptosis [28].

In this study, we hypothesize that inhibition of HSP70 by quercetin would exacerbate intestinal injury and the involvement of MAPK pathway in this effect is expected. Thus, quercetin was applied in present study to examine the role of HSP70 expression on activation of the MAPK pathway in the small intestine of heat stressed rats.

## 2. Materials and Methods

### 2.1. Animals

All experimental procedures and protocols used in this study were reviewed and approved by the Animal Care and Use Committee of the Peking Union Medical College (Beijing, China) and conformed to the Guide for the Care and Use of Laboratory Animals published by the US National Institutes of Health (revised in 1996). Forty-eight 6-week-old healthy male Sprague-Dawley (SD) rats weighing 200±10 g were obtained from the Vital River Experimental Animal Company (Beijing, China). Three rats were housed in a plastic cages 25 × 40 cm and 25 cm height in a temperature-controlled room controlled at 23°C and 55% relative humidity (RH) under a 12/12 h light/dark cycle, with ad libitum access to food and water and humane care. The body weight was determined at the beginning and indicated time to calculate the average daily gain. All animal experiments were performed by qualified researchers certified by the Beijing Association on Laboratory Animal Care (Beijing, China).

### 2.2. Grouping

After a 7-day acclimation period, the rats were randomly divided into three groups: control (CON), heat stress (HS), and heat stress + quercetin (HQ) (n=16/group). Rats in the CON group were housed under normal conditions (23°C, 55% RH) throughout the experimental period.

The HS and HQ rats were housed under the same conditions as the CON group but were exposed to environmental heat stress (43°C for 50 min) in a heating chamber without anesthesia between 1000 am and 1100 am for 14 consecutive days. During heating, rats had free access to food and water. After heating, all rats were immediately returned to normal housing conditions.

The rats in HQ group were first dosed with 0.5 mL of quercetin (50 mg/kg body weight) [29, 30] via intragastric gavage at 0900 am daily and then exposed to heat stress as described above. The CON and HS groups were gavaged with 0.5 mL saline using the same schedule. On day 7 and day 14, eight rats from each group were sacrificed by decapitation without anesthesia immediately following the final heat exposure.

### 2.3. Sample Collection

Following euthanize, blood was collected through the inferior vena cave vein of each rat at about 1100 am and centrifuged at 3000 g (10 min, 4°C) to obtain serum. The serum was stored in 1-mL Eppendorf tubes at -80°C until use. Segments of the jejunum (5 cm posterior to the yolk stalk) were removed and thoroughly flushed with physiological saline (0.9%) to remove all intestinal contents. Then, the jejunum was dissected and divided into two parts: a 2-cm segment was fixed in 10% buffered formalin phosphate for histological analysis; the rest was scraped with glass slides to remove the whole mucosa and was immediately frozen in liquid nitrogen and preserved at -80°C for western blot analysis.

### 2.4. Measurement of Rectal Temperature

Rectal temperature was recorded at 0200 pm on days 1, 7, and 14 after heat stress, using a digital clinical thermometer (model “mini color”; ICO Technology, Barcelona, Spain; range, 32-43.9°C; accuracy, ±0.1°C). For each rat, averages of rectal temperatures were obtained from three independent measures on each day [31].

### 2.5. Measurement of Blood Constituents

Serum cortisol levels were determined using a radioimmunoassay (RIA): Iodine^[125I]^ Cor RIA kit (Beijing Chemclin Biotech Co., Ltd., Beijing, China), according to the manufacturer's instructions using an Automatic *γ* Immune instrument (DFM-96, Hefei Zhongcheng Mechanical & Electrical Co., China). The inter- and intra-assay coefficients of variation were <9.5 and <7.5%, respectively. Serum D-lactate (D-LAC) and diamine oxidase (DAO) levels, which are the markers of intestinal permeability damage, were tested by ELISA (Immundiagnostik, Bensheim, Germany), according to the manufacturer's instructions.

### 2.6. Morphological Examination

Fixed jejunal samples were prepared using conventional paraffin embedding techniques. Samples were sectioned at 6 *μ*m thick and stained with hematoxylin and eosin. Villus height and crypt depth were measured according to Wu et al. [32] under a light microscope (CK-40, Olympus, Tokyo, Japan). A total of 10 intact, well-oriented, crypt-villus units were measured in triplicate for each intestinal cross-section. Villus height was measured from the tip of the villus to the villus-crypt junction; crypt depth was defined as the depth of the invagination between adjacent villi. The histological analysis was performed by an investigator who was unaware of the origin of tissue sections.

### 2.7. Western Blot

The jejunal mucosal samples were minced and placed in lysis buffer containing protease inhibitors (Roche Applied Science, Penzberg, Germany) and phosphatase inhibitors (Roche Applied Science, Penzberg, Germany). Samples were homogenized for 30 s and centrifuged at 13,000 g for 20 min at 4°C to remove any insoluble debris. Protein concentrations were analyzed using the BCA reagent (Beijing SiNoble Biotechnology Inc., Beijing, China). Aliquots of 20 *μ*g protein were loaded and separated by SDS-PAGE and transferred onto a 0.45-*μ*m nitrocellulose membrane. After blocking for 30 min in 3% BSA-TBST, the membranes were incubated with the primary antibody for 3 h at room temperature or overnight at 4°C. The primary antibodies used were HSP70 (1 : 2000, TDYBio, Beijing, China), phospho-ERK1/2 (1 : 2000, Cell Signaling Technology, Danvers, MA, USA), ERK1/2 (1 : 1000, Cell Signaling Technology, Danvers, MA, USA), phospho-p38 and p38 (1 : 1000, Cell Signaling Technology, Danvers, MA, USA), phospho-JNK and JNK (1 : 1000, Cell Signaling Technology, Danvers, MA, USA), procaspase-3 and cleaved caspase-3 (1 : 2000, Cell Signaling Technology, Danvers, MA, USA), and GAPDH (1 : 20000, TDYBio, Beijing, China). The washed membranes were incubated for 40 min at room temperature with horseradish peroxidase-conjugated secondary antibodies. The membranes were washed and incubated with an enhanced chemiluminescence substrate (ECL; Thermo Fisher Scientific, Waltham, MA, USA). The antibody-specific protein bands were visualized with an electrochemiluminescence substrate using a gel imaging system (Tanon Science and Technology, Shanghai, China) with the Image Analysis Software (National Institutes of Health, Bethesda, MD, USA).

### 2.8. Statistical Analysis

All statistical analyses were performed using SAS 8.2 (SAS Institute, Cary, NC, USA). All data were expressed as means ± standard error of mean (SEM). Statistical analyses were performed by one-way analysis of variance (ANOVA) followed by the Duncan multiple range tests, when appropriate. Two-sided P-values <0.05 were considered statistically significant.

## 3. Results

### 3.1. Effect of Heat Stress and HSP70 Inhibition on Body Weight

**Figure 1:**
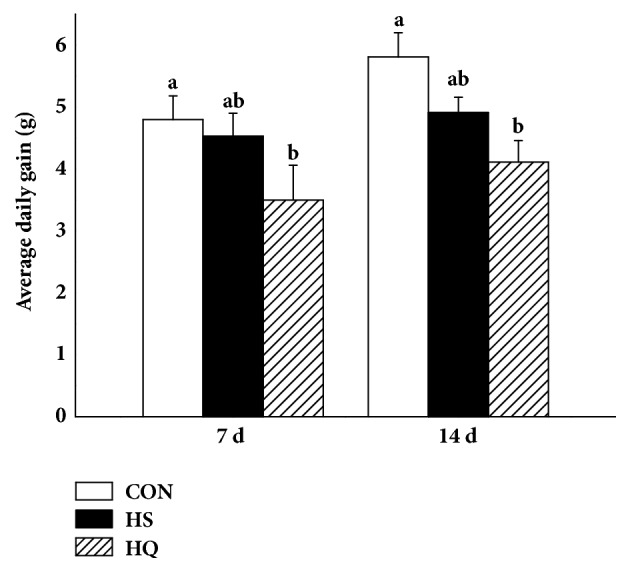
Effects of HSP70 inhibition on the body weight of heat stressed rats. The experiment lasted for 14 days with daily 50 min of heat stress treatment (43°C) for the HS and HQ groups. Rats of HQ group were intragastrically given 0.5 mL quercetin solution (50 mg/kg body weight) before the heat stress treatment. Data are shown as means ± standard error of mean (SEM) (n=8). The values with different letters (a, b) are significantly different (P<0.05). CON: control; HS: heat stress; HQ: heat stress + quercetin.

The effects of the heat stress and quercetin treatments on body weight of rats are shown in Figure 1. Body weight of the HQ group was significantly lower (P<0.01) compared with the CON group on both sampling days (days 7 and 14). No differences were observed between other groups. These results indicated that HSP70 expression had no significant effect on body weight under heat stress.

### 3.2. Effect of Heat Stress and HSP70 Inhibition on Heat Stress Indexes

**Figure 2:**
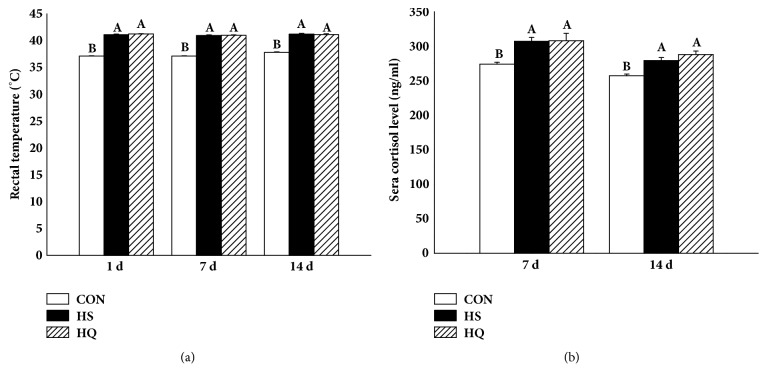
Effects of HSP70 inhibition on rectal temperature (a) and serum cortisol level (b) of heat stressed rats. The experiment lasted for 14 days with daily 50 min of heat stress treatment (43°C) for the HS and HQ groups. Rats of HQ group were intragastrically given 0.5 mL quercetin solution (50 mg/kg body weight) before the heat stress treatment. (a) Rectal temperature was recorded on days 1, 7, and 14 of heat stress. (b) Serum cortisol levels on days 7 and 14 of heat stress were determined using a radioimmunoassay. Data are shown as mean ± standard error of mean (SEM) (n=8). The values with different letters (A, B) are significantly different (P<0.05). CON: control; HS: heat stress; HQ: heat stress + quercetin.

Rectal temperature was used to assess heat stress. Following high-temperature treatment, rat rectal temperature was noticeably elevated in the HS and HQ groups on days 1, 7, and 14 compared with CON rats (P<0.001), but there was no difference between the HS and HQ groups (Figure 2(a)).

Another biomarker for the evaluation of heat stress is serum cortisol. Compared with the CON group, the cortisol level in the HS group and the HQ groups was significantly higher (P<0.05) on days 7 and 14. No difference was observed between the HS and HQ groups (Figure 2(b)). These data indicated that there were no significant effects of HSP70 expression on heat stress parameters.

### 3.3. Effect of Heat Stress and HSP70 Inhibition on Jejunum Morphology

**Figure 3:**
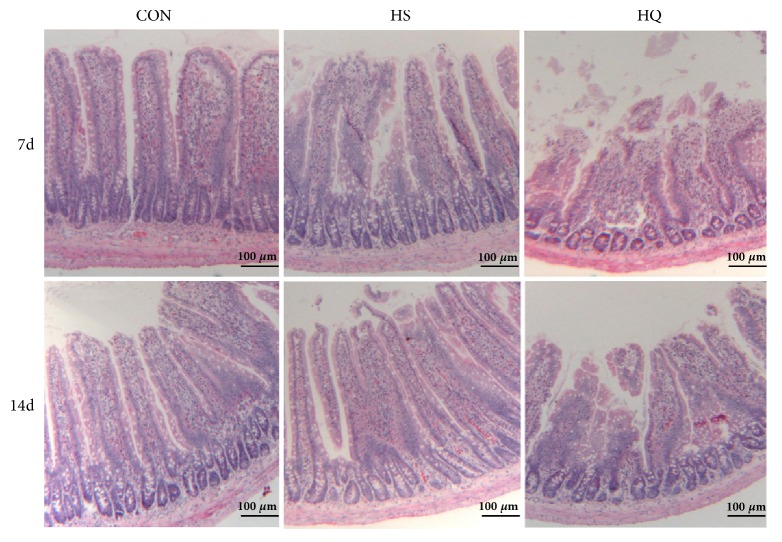
Effects of HSP70 inhibition on the jejunal morphology of heat stressed rats. The experiment lasted for 14 days with daily 50 min of heat stress treatment (43°C) for the HS and HQ groups. Rats of HQ group were intragastrically given 0.5 mL quercetin solution (50 mg/kg body weight) before the heat stress treatment. The jejunum from rats in the CON, HS, or HQ groups on days 7 and 14 of heat stress was examined by hematoxylin-eosin staining. Scale bar = 100 *μ*m. CON: control; HS: heat stress; HQ: heat stress + quercetin.

To examine the effects of HSP70 expression on small intestinal morphology under heat stress, jejunal sections stained with hematoxylin-eosin were observed by light microscopy. Regularly aligned microvilli and integral mitochondria were observed in the jejunal cells of CON samples. In contrast, the microvilli were markedly damaged in the HS rats, and the injury was worse by quercetin treatment (HQ group) (Figure 3). Furthermore, there were significant differences in villus height between groups on day 7 (CON: 534 versus HS: 469 versus HQ: 407 nm, all P<0.05). On day 14, difference in villus height was only found between the CON and HQ groups (CON: 561 versus HQ: 428 nm, P=0.04). No significant differences were observed in crypt depth or villus height: crypt ratio on days 7 and 14 (all P>0.05) (Table 1).

### 3.4. Effect of Heat Stress and HSP70 Inhibition on Jejunum Permeability

**Figure 4:**
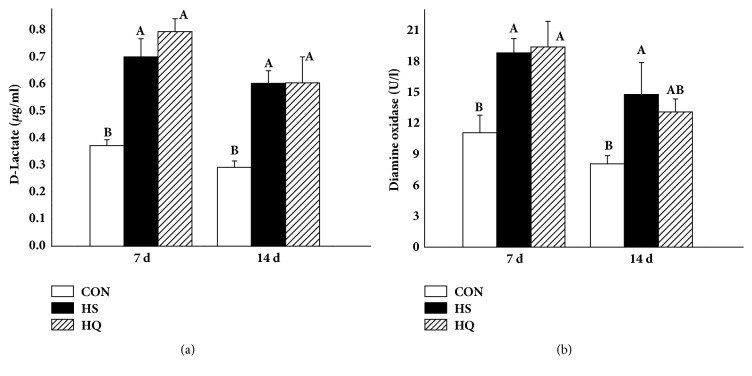
Effects of HSP70 inhibition on the intestinal integrity and function of heat stressed rats. The experiment lasted for 14 days with daily 50 min of heat stress treatment (43°C) for the HS and HQ groups. Rats of HQ group were intragastrically given 0.5 mL quercetin solution (50 mg/kg body weight) before the heat stress treatment. (a) D-lactate and (b) diamine oxidase levels were detected by ELISA on days 7 and 14 after heat stress. Data are shown as mean ± standard error of mean (SEM) (n=8). The values with different letters (A, B) are significantly different (P<0.05). CON: control; HS: heat stress; HQ: heat stress + quercetin.

Serum D-LAC and DAO levels were measured as markers of intestine integrity (Figure 4). Heat stress resulted in an increase in mucosal permeability characterized by serum D-LAC and DAO levels (P<0.05). There was no significant difference in the levels of D-LAC and DAO between the HS and HQ groups, suggesting that inhibition of HSP70 did not affect the intestinal mucosal permeability.

### 3.5. Effect of Heat Stress and HSP70 Inhibition on the MAPK Pathway

**Figure 5:**
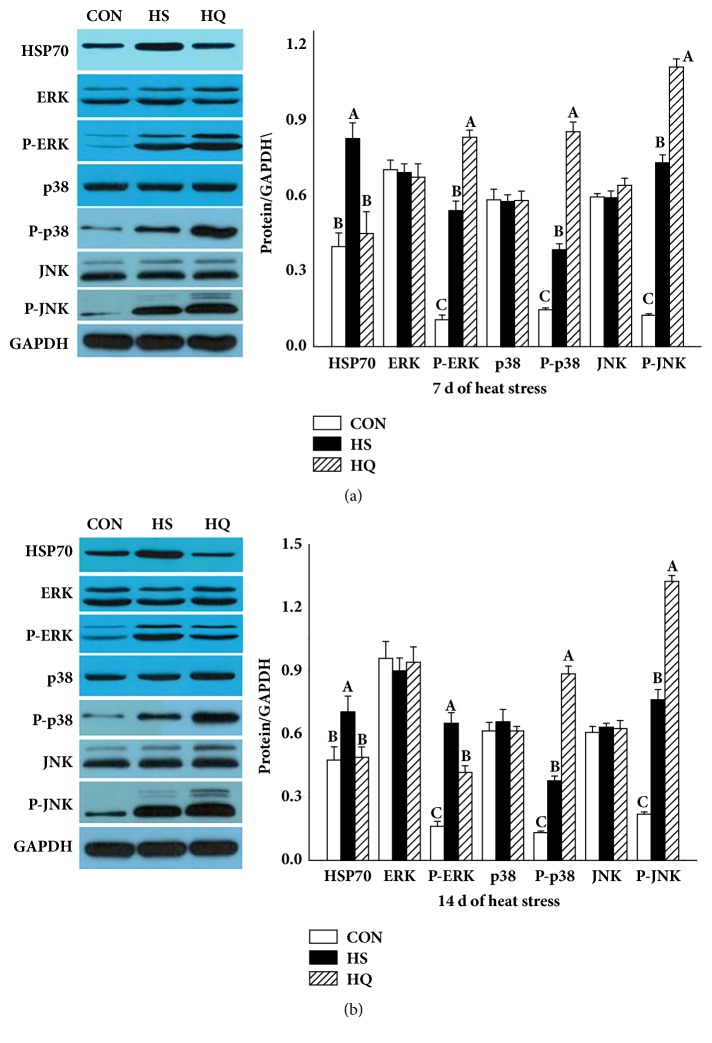
Effects of HSP70 inhibition on the HSP70 expression and activation of MAPK pathway in the jejunal mucosa of heat stressed rats. The experiment lasted for 14 days with daily 50 min of heat stress treatment (43°C) for the HS and HQ groups. Rats of HQ group were intragastrically given 0.5 mL quercetin solution (50 mg/kg body weight) before the heat stress treatment. Western blotting was used to detect the protein levels of MAPK. (a) On day 7 of heat stress. (b) On day 14 of heat stress. Data are shown as mean ± standard error of mean (SEM) (n=6). The values with different letters (A, B, C) are significantly different (P<0.05). CON: control; HS: heat stress; HQ: heat stress + quercetin.

To investigate the role of HSP70 expression on activation of the MAPK pathway during heat stress, the MAPK protein kinases ERK, p38MAPK, and JNK were measured by western blot (Figure 5). Compared with the CON group, the levels of HSP70, phospho-ERK, phospho-p38, and phospho-JNK were significantly higher (P<0.001) in the HS group on days 7 and 14. Compared with the HS group, the levels of HSP70 in the HQ group were significantly lower (P<0.001), but the levels of phospho-ERK, phospho-p38, and phospho-JNK were higher (P<0.001) in the HQ group on day 7. Although the levels of phospho-p38 and phospho-JNK in the HQ group were significantly higher than that of the HS group on day 14, the levels of phospho-ERK in the HQ group were significantly lower than that of the HS group. These results suggested that heat stress induced an increase of HSP70 and the activation of the MAPK pathway, and inhibition of HSP70 expression affected the MAPK signaling pathways.

### 3.6. Effect of Heat Stress and HSP70 Inhibition on Activation of Caspase-3

**Figure 6:**
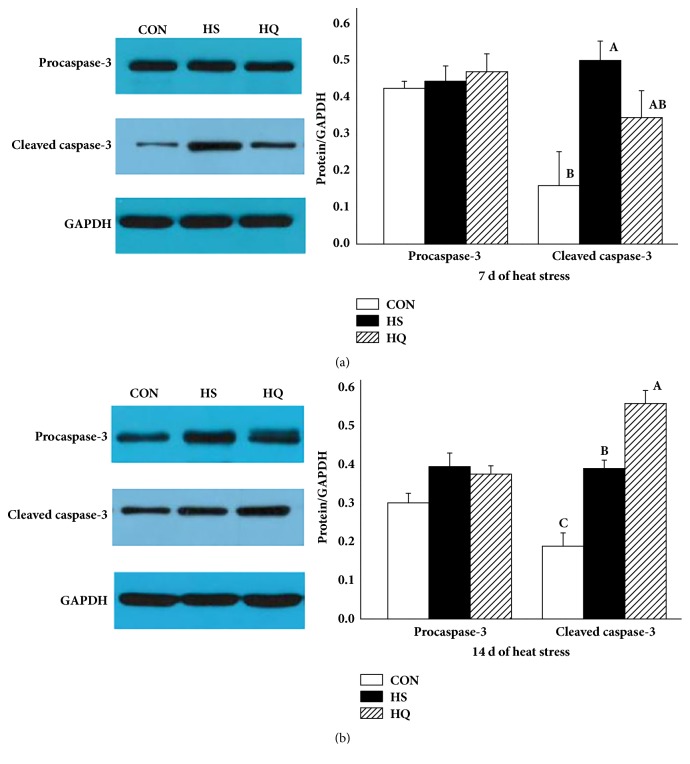
Effects of HSP70 inhibition on the activation of caspase-3 in the jejunal mucosa of heat stressed rats. The experiment lasted for 14 days with daily 50 min of heat stress treatment (43°C) for the HS and HQ groups. Rats of HQ group were intragastrically given 0.5 mL quercetin solution (50 mg/kg body weight) before the heat stress treatment. Western blotting was used to detect the protein levels of procaspase-3 and cleaved caspase-3. (a) On day 7 of heat stress. (b) On day 14 of heat stress. Data are shown as mean ± standard error of mean (SEM) (n=6). The values with different letters (A, B, C) are significantly different (P<0.05). CON: control; HS: heat stress; HQ: heat stress + quercetin.

To determine whether HSP70 protects cells from heat stress-induced apoptosis, we investigated the expression of apoptosis-related protein in the jejunal mucosa (Figure 6). Compared with the CON group, the levels of cleaved caspase-3 were significantly higher (P<0.001) in the HS group on days 7 and 14, but there were no significant differences in the levels of procaspase-3 between the two groups. On day 7 of heat stress, the levels of procaspase-3 and cleaved caspase-3 in the HQ group were similar to the levels in the HS group. On day 14 of heat stress, the levels of cleaved caspase-3 in the HQ group were significantly higher than that of the HS group (P<0.001). These data indicated that the inhibition of apoptosis by HSP70 mainly occurred in the late stage of heat stress.

## 4. Discussion

Heat stress is an important factor influencing domestic animal production during in summer months [33, 34]. HSP70 is a protein involved in modulating a variety of physiological processes, including proliferation, apoptosis, and stress responses. Hence, in this study, the expression of HSP70 protein was inhibited by quercetin to explore its potential role in protecting against heat stress-induced injury in the small intestine of rats.

Rectal temperature and plasma cortisol were higher in the HS and HQ groups compared to the CON group. The alterations in these parameters, commonly considered as an indication of heat stress [6, 35], indicated that the rats experienced moderate hyperthermia. There were no obvious differences in rectal temperature and plasma cortisol between the HS and HQ groups, which indicated that inhibition of HSP70 expression did not affect these parameters.

The intestine is susceptible to heat stress, hypoxia, and other environmental factors, resulting in mucosal damage [4–6]. In our morphological study, we also found that heat treatment over 7 days caused marked damage to the tips of the intestinal villi, e.g., inducing epithelial cell shedding, exposing the intestinal mucosa lamina propria, and shortening villus height. Inhibiting HSP70 with quercetin aggravated mucosal damage (as shown by villus height) at 7 days compared with heat shock alone, but the difference disappeared on day 14.

Heat stress leads to increased intestinal permeability in various mammalian species [36–38]. The serum D-LAC and DAO levels are recognized as sensitive markers for monitoring the alteration of intestinal barrier permeability [3, 39]. In the current experiment, D-LAC was increased during heat stress, suggesting that the intestinal barrier function was compromised. In addition, the content of DAO in the sera was also significantly increased after heat stress treatment. Inhibiting HSP70 with quercetin did not increase intestinal permeability compared with heat shock alone.

Thermal stress in the form of hyperthermia has been shown to stimulate the phosphorylation and thus activation of multiple MAPKs, a superfamily of protein kinases [6, 14]. In mammals, three primary MAPKs are found, including extracellular signal-regulated protein kinase (ERK), c-Jun NH2-terminal kinase (JNK), and p38 MAPK [40]. He et al. [4] showed that heat stress-induced injury in rat small intestine activated the MAPK signaling pathways. In this study, we also found p38MAPK, JNK, and ERK exhibited considerable phosphorylation profiles in samples from jejunum tissue of rats exposed to hyperthermia.

Recent reports indicate that broad cytoprotective functions of HSP(s), particularly the HSP70 family, are associated with MAPK signaling [14, 41]. It is shown that HSP70 can inhibit Bax oligomerization [42], which might be due to its ability to inhibit JNK activation [43]. Increased HSP70 may also reduce the activation of p38 kinase [10]. For this reason, we investigated the involvement of HSP70 in the induction of MAPKs and the mechanism of protection of intestine after heat stress. Our results indicate that the phosphorylation of three MAPK signal proteins was significantly influenced by the level of HSP70. Indeed, in the HS group, HSP70 was increased as expected, and phospho-ERK, phospho-p38, and phospho-JNK were increased, probably as an effect of thermal stress. When inhibiting HSP70 with quercetin, the protective effect of HSP70 was abrogated, leading to further changes in phospho-ERK, phospho-p38, and phospho-JNK. These results are supported by previous studies which reported that there was a correlation between protective HSP70 expression and activation of the MAPK pathway [20, 21]. Bironaite et al. [14] showed that the activation of JNK1, JNK2, and p38 was necessary for HSP70 induction and cell protection. Inhibition of ERK1/2 exacerbated the effects of heat stress on the intestine, while inhibiting JNK or p38 attenuated the effects of the stress [44]. Nevertheless, whether the increased MAPK signaling observed in the present study is caused by HSP70 inhibition or a cellular compensatory mechanism trying to increase HSP70 expression is still unknown.

HSPs are well-known mediators in response to thermal stress and are involved in regulating cell survival under various environmental and physiological stresses [45, 46]. JNK and p38 activities are in most cases associated with promotion of apoptosis, whereas ERK activity is generally associated with protection [44, 47]. In the present study, inhibition of HSP70 expression significantly activated three MAPK signal pathways on day 7 of heat stress. Nevertheless, there was no significant difference in caspase-3 activation between the HS and HQ groups on day 7 of heat stress. On the contrary, inhibition of HSP70 expression significantly activated the JNK pathway, inhibited the phosphorylation of ERK, and increased caspase-3 activation on day 14 of heat stress. This suggests that HSP70 may play an intestine protective role and that the MAPK signaling pathways are involved in this process, but the exact roles of each molecular in the MAPK signaling pathways are worth being examined.

## 5. Conclusion

The present study showed that heat stress could result in decreased intestinal integrity and function, increased HSP70 expression, and activated MAPK signaling. The results also suggested that the MAPK signaling was involved in the protective role of HSP70 against heat stress-induce injury in the small intestine of rats. Thus, this study provides further insights into the intestinal protective mechanisms of HSP70, which could reveal novel targets for the protection of the intestine in the context of heat stress.

## Figures and Tables

**Table 1:** Effects of different treatments on jejuna villus height, crypt depth, and V/C of rats.

Day	Index	CON	HS	HQ	P
7	Villus height (nm)	533.5^a^	468.7^b^	406.9^c^	<0.01
	Crypt depth (nm)	159.7	157.4	150.3	0.80
	V/C	3.4	3.1	2.8	0.13
14	Villus height (nm)	560.5^a^	457.5^ab^	428.0^b^	0.04
	Crypt depth (nm)	166.9	181.1	161.6	0.36
	V/C	3.4^a^	2.6^b^	2.7^b^	0.05

CON: control; HS: heat stress; HQ: heat stress + quercetin.

^a, b, c^Values with different letters are significantly different (P<0.05).

## Data Availability

The data used to support the findings of this study are available from the corresponding author upon request.
